# Muscle Length of the Hamstrings Using Ultrasonography Versus Musculoskeletal Modelling

**DOI:** 10.3390/jfmk6010026

**Published:** 2021-03-12

**Authors:** Eleftherios Kellis, Athina Konstantinidou, Athanasios Ellinoudis

**Affiliations:** Laboratory of Neuromechanics, Department of Physical Education and Sport Sciences at Serres, Aristotle University of Thessaloniki, 62100 Serres, Greece; athinakonstandi@hotmail.gr (A.K.); ellinoud@phed-sr.auth.gr (A.E.)

**Keywords:** extended-view ultrasonography, biceps femoris long head, semitendinosus, semimembranosus, architecture, modelling

## Abstract

Muscle morphology is an important contributor to hamstring muscle injury and malfunction. The aim of this study was to examine if hamstring muscle-tendon lengths differ between various measurement methods as well as if passive length changes differ between individual hamstrings. The lengths of biceps femoris long head (BFlh), semimembranosus (SM), and semitendinosus (ST) of 12 healthy males were determined using three methods: Firstly, by identifying the muscle attachments using ultrasound (US) and then measuring the distance on the skin using a flexible ultrasound tape (TAPE-US). Secondly, by scanning each muscle using extended-field-of view US (EFOV-US) and, thirdly, by estimating length using modelling equations (MODEL). Measurements were performed with the participant relaxed at six combinations of hip (0°, 90°) and knee (0°, 45°, and 90°) flexion angles. The MODEL method showed greater BFlh and SM lengths as well as changes in length than US methods. EFOV-US showed greater ST and SM lengths than TAPE-US (*p* < 0.05). SM length change across all joint positions was greater than BFlh and ST (*p* < 0.05). Hamstring length predicted using regression equations is greater compared with those measured using US-based methods. The EFOV-US method yielded greater ST and SM length than the TAPE-US method. SM showed the highest change in length at different hip and knee joint positions.

## 1. Introduction

Hamstring muscle injuries are frequent in sport and they can have a serious impact on an athlete’s performance and career [1]. In addition, hamstring malfunction is linked with pathological conditions such as anterior cruciate ligament injury [2], low back pain [3], or spasticity [4]. Hamstring anatomy and morphology is considered an important contributor to such injuries [2,3] or rehabilitation using surgical lengthening [4]. For this reason, studies have focused on a detailed anatomy of this muscle group [5,6,7].

The hamstring muscle group includes the semimembranosus (SM), the semitendinosus (ST), and the long head of the biceps femoris (BFlh). Although these muscles act as hip extensors and knee flexors, research has clearly indicated that there are various differences in anatomy and architecture between them [5,6,7]. Muscle-tendon unit length (LMTU) is an important architecture parameter as it may influence the mechanical properties of each muscle, such as the resistance to stretch and contractile function. Cadaveric studies have shown that ST has a greater muscle-tendon unit length than SM and BFlh [8,9,10,11,12]. This may indicate that for the same change in knee and hip position, each hamstring component may display a different change in length. For this reason, improvement of the methods used to estimate hamstrings LMTU may assist in a better understanding of the mechanical properties of this particular muscle group.

A popular method for the quantification of LMTU is the use of regression anthropometric equations [8,9,13] (MODEL method). These equations are based on actual measurements of the joint angle and the LMTU from cadavers. Hence, this method allows a quantification of the changes in hamstring LMTU during dynamic movement as a function of the changes in hip and knee joint angles. Such equations are frequently incorporated into mathematical simulation models that are used to predict hamstring muscle kinematics and loading during dynamic movements [14,15,16,17]. However, a limitation of this technique is that it is based on data obtained in cadavers and, hence, the obtained muscle lengths do not take individual variability in morphology into consideration.

To measure LMTU, the actual distance between the attachments along the muscle path is required. Some studies have used ultrasound (US) to quantify hamstring LMTU in vivo [10,18]. In particular, the LMTU was measured using a tape measure directly on the skin after identifying the proximal and distal attachments of the hamstrings using US (TAPE-US method). Muscle length measured with this method displays a high correlation with cadaveric data [19] allowing for a direct assessment of individual hamstring lengths in vivo. However, variations in the muscle path that is drawn along the skin surface and tissue morphology underneath the skin may limit its applicability.

Extended field-of-view US (EFOV-US method) is another method for the examination of muscle-tendon architecture [20,21]. This technique uses an algorithm to automatically fit a series of images, allowing for the scanning of entire tissue areas within one continuous scan [20]. The validity and reliability of this technique for has been previously reported in quadriceps [21] and hamstrings [22]. Although EFOV is a two-dimensional imaging technique, it may be particularly useful for an easy, accurate, and reliable measurement of long muscle lengths in vivo [20]. To our knowledge, no study has attempted the measurement of the whole LMTU of hamstring muscles using EFOV.

Very few studies have reported actual LMTU values as a function of the hip and knee joint angle [9,11,12]. Specifically, knee extension was associated with an increase in hamstring LMTU, but the amount of length change differs between individual hamstring components. In absolute terms, the highest LMTU change is displayed by the ST, followed by the SM, the BFlh, and finally the BFsh [9,11,12]. However, these results were mainly obtained from cadavers [12] or estimated using geometric modelling techniques [11]. Some studies have quantified LMTU using three-dimensional free-hand ultrasound [23,24] but these studies did not compare LMTU between different hamstring components. Although three-dimensional measurements offer more in-depth details of the muscle-tendon architecture [23,24], simpler methods, such as TAPE-US or EFOV may provide an easier and a more time-efficient evaluation of muscle-tendon lengths. To our knowledge, differences between individual hamstring components in either resting length or in change in length due to joint motion have not been quantified using US in vivo.

Developing methods for the in vivo quantification of hamstring morphology allows an in depth understanding of their mechanical properties during dynamic conditions. The use of regression equations may provide estimates of LMTU but they are based on cadaveric measurements. US techniques allow for a direct assessment of LMTU that is specific to the characteristics of an individual. To the best of our knowledge, there is not a universally acceptable technique to measure hamstring muscle length. Furthermore, it is currently unclear whether LMTU differs between various measurement methods. If LMTU measurement is method-dependent, comparisons of hamstring length-related properties between different studies is questionable. In addition, no study has examined whether LMTU quantified using the simpler TAPE-US method differs in comparison to the more advanced EFOV-US method. The purpose of the present study was to compare the LMTU of BFlh, SM, and ST measured using three techniques: TAPE-US, EFOV-US, and MODEL. A secondary aim was to examine differences in LMTU between hamstrings at various combinations of hip and knee flexion angles. We tested three hypotheses: First, that LMTU estimated using the MODEL method would differ compared that measured using US, second, that LMTU would differ between US-TAPE and EFOV-US techniques, and third, that the LMTU change during various hip and knee joint positions would differ between individual hamstring muscles.

## 2. Materials and Methods

### 2.1. Participants

A total of 12 males (mean (SD) age 24.9 (0.3) years; mass 81.5 (2.3) kg; height 1.77 (0.02) m) volunteered to participate in this study after signing written informed consent. The participants were healthy and had no injury of the lower limbs including a history of hamstring strain or any other muscle or ligamentous injury of the knee. The participants were physically active, but they did not engage in a specific sport or exercise program during the measurement period. The procedures conformed with the Declaration of Helsinki and were approved by the university ethics review committee (ERC 006/2020).

### 2.2. Experimental Design

The participants were instructed to refrain from any vigorous activity for 24 h before the testing visits. During each visit, joint angles and US images of the BFLH, ST, and SM were taken to quantify LMTU using TAPE-US, EFOV, and MODEL methods.

### 2.3. Ultrasound Assessment

Ultrasound images of the hamstrings were obtained using a portable brightness mode (B-mode) ultrasound imaging device (GE Logiq e, Wauwatosa, WI, USA) and a multifrequency linear-array probe (12 L-RS, 5–13 MHz, and 40.0-mm field-of-view).

**Figure 1:**
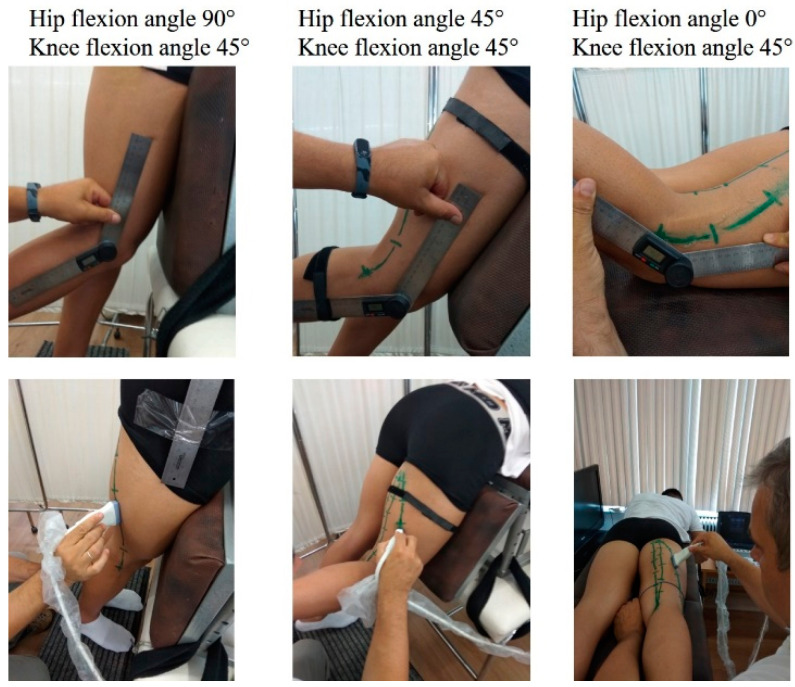
(Upper panel) Illustration of the position of the participant at three characteristic combinations of knee and joint positions. In each position the knee and hip joint angle were measured using an analogue goniometer. (Lower panel) Example of ultrasound scanning along the muscle path in each joint configuration.

**Figure 2:**
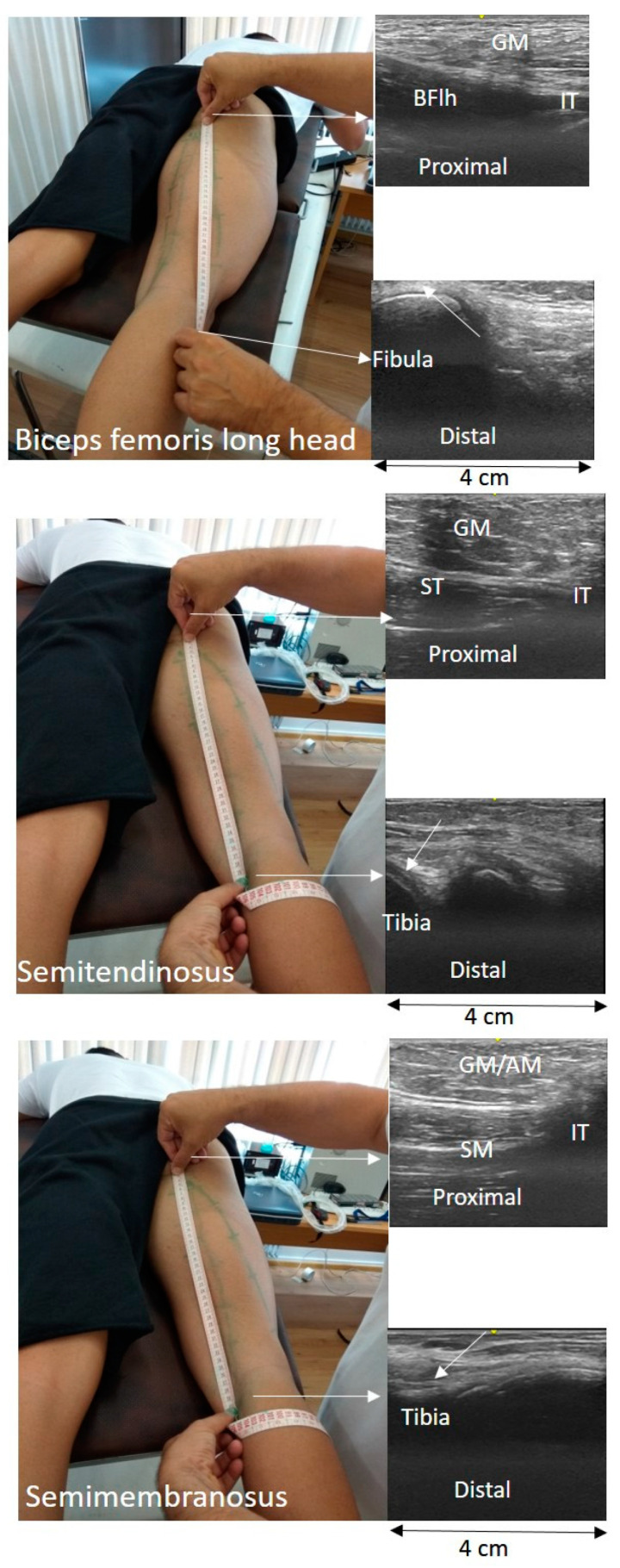
Measurement of muscle-tendon unit length using the tape-ultrasound (TAPE-US) technique. In each testing position, the distal origin and proximal ends of the biceps femoris long head (BFlh), semitendinosus (ST), and semimembranosus (SM) were first identified and marked on the skin (arrows indicate tendon origin). The path of the muscle-tendon unit was drawn on the skin surface with the assistance of US images obtained as the probe moved slowly from the proximal to the distal end. Subsequently, the distance between the two ends was measured using a tape measure (GM = gluteus maximus, AM = adductor magnus, and IT = ischial tuberosity).

The TAPE-US protocol included a measurement of the distance between the muscle attachments on the skin (Figure 1). In particular, in each testing position, the BFlh, ST, and SM distal and proximal attachments were first identified using B-mode scans and marked on the skin. For all muscles, the proximal attachment was the ischial tuberosity. The insertion of the BFlh tendon at the fibular head was considered as its distal attachment. The distal origin of the SM was the posterior surface of the medial condyle of the tibia. Changes in ST free tendon at the remaining joint positions were measured on the skin surface with a tape measure. Subsequently, the US probe moved slowly from the distal to the proximal end of the muscle and the muscle-tendon unit path was marked on the skin. A flexible tape was placed along the skin from the proximal to the distal marker. The distance between the two ends was measured using the tape (to the nearest mm) and it was considered as the whole LMTU (Figure 2).

**Figure 3:**
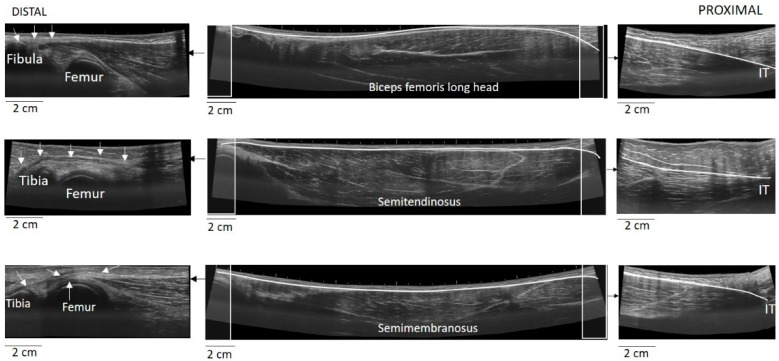
Measurement of muscle-tendon unit using EFOV-US technique. In each panel of images, the center image presents each muscle-tendon unit, scanned from the distal to proximal end. To better illustrate the identification of the attachments, zoomed EFOV-US images of the distal (left side) and proximal (right side) are also included. White arrows illustrate the path of each distal tendon. Using the segmented line tool of the ImageJ software, the superficial aponeurosis was digitised and the total line length was considered as the muscle-tendon unit (IT = ischial tuberosity).

The EFOV protocol included continuous scanning of the whole LMTU from the distal to proximal end (Figure 3). In each testing position, the US probe was slowly and continuously moved from the most proximal to the most distal aspect of each muscle. The probe was moved perpendicularly to the skin. To achieve better and consistent images, each muscle was scanned and the path of the probe that best identified the underlying muscle was marked on the skin. Subsequently, each MTU was scanned until three scans with acceptable image quality (i.e., a clear, continuous depiction of the superficial and deep aponeurosis without any sudden changes in the image across the muscle) were obtained. On several occasions, the distal and proximal tendons could not be visualised using a single long scan due to uneven surface anatomy. In these cases, we used an echo-absorptive marker on the skin and additional EFOV scans of these muscle segments were taken. The long free tendon of the ST from the medial aspect of the tibia to the fascia cruris was evaluated only with the hip in neutral position and the knee at 45° of the flexion, as due to curvature of the tendon along the femur and the tibia, complete visualisation of its path at all combinations of hip and joint movement was difficult. Great care was taken to ensure that consistent minimal pressure was applied with the probe to avoid compression of the muscle. Furthermore, a generous amount of water-soluble transmission gel was applied to the skin, to enhance acoustic coupling and reduce near-field artifacts. GE Logiq LogicView software was used to produce panoramic images of each MTU in real time. The US EFOV images were digitally stored and analysed using ImageJ Software (Version 1.47v, National Institutes of Healt, Bethesda, MD, USA). In each image, the segmented line tool was used to digitise the LMTU from the most proximal to most distal end of the superficial aponeurosis (Figure 3). In cases where more than one scan was used to visualise the same muscle, the final LMTU was estimated by adding the lengths of the muscle in each respective scan.

### 2.4. Prediction Using Regresion Equations

The MODEL method consisted of estimation of LMTU (from angular position data using the regression Equations (1)–(3), as provided by Hawkins and Hull [8]:

(1) $${\mathrm{LMTU}}_{\mathrm{BFlh}}=1.048+0.00209\cdot \hat{\theta }-0.00160\cdot \hat{\varphi }$$

(2) $${\mathrm{LMTU}}_{\mathrm{ST}}=0.987+0.00207\cdot \hat{\theta }-0.00178\cdot \hat{\varphi }$$

(3) $${\mathrm{LMTU}}_{\mathrm{SM}}=1.027+0.00199\cdot \hat{\theta }-0.0222\cdot \hat{\varphi }$$

where LMTU_BFlh_, LMTU_ST_, and LMTU_SM_ are the lengths of BFlh, ST, and SM, respectively, as percentages of thigh length, and $\hat{\theta }$, $\hat{\varphi }$ are hip and knee joint angles in degrees, with the anatomical position being zero. Thigh length was measured in each participant, from the great trochanter to lateral femoral condyle, and it was subsequently used to calculate absolute LMTU in cm.

In a pilot study, 7 participants were retested one week after the main testing session by the same operator. Reliability statistics include the intraclass correlation coefficient (ICC_2,1_) as well as the standard error of measurement (SEM) which was calculated as $\mathrm{SEM}=\mathrm{SD}\sqrt{1-\mathrm{ICC}}$, where SD is the standard deviation of the differences between test and retest values. The results indicated that the ICC ranged from 0.85 to 0.95 and the SEM from 1.87% to 4.63% for the TAPE-US method (Table 1). The EFOF-US method measurements showed an ICC range from 0.84 to 0.99 and a SEM ranging from 1.44% to 2.53% (Table 1).

For each muscle and hip joint configuration, absolute LMTU values (in cm) were further analysed. In addition, the maximum percentage change of LMTU across the 6 testing conditions relative to the shortest muscle length (hip flexion angle 0° and knee flexion angle 90°) was also estimated.

### 2.5. Statistical Analysis

For each muscle, three-way analysis of variance (ANOVA) designs were used to examine the effects of the method (3 levels), hip (2 levels), and knee (3 levels) joint positions on absolute LMTU values. The maximum percentage change in LMTU was compared between methods and muscles using a two-way ANOVA. Effect sizes (η^2^) were also monitored and significant interactions were followed up with simple effects tests and, if significant, post-hoc Tukey tests were applied to examine significant differences between pairs of means. Statistical significance was set at *p* < 0.05.

## 3. Results

For all ANOVA designs, the three-way interaction effect on absolute LMTUs was non-statistically significant (*p* > 0.05).

The average BFlh LMTU for all combinations of hip and knee joint positions is presented in Table 2. The ANOVA showed statistically significant main effects for method (F_2,22_ = 32.57, *p* < 0.05. η^2^ = 0.75), hip (F_1,11_ = 620.77, *p* < 0.05, η^2^ = 0.98), and knee (F_2,22_ = 763.81, *p* < 0.05, η^2^ = 0.98). Post-hoc Tukey tests showed that LMTU estimated using EFOV-US and TAPE-US methods were significantly lower compared with the MODEL method (*p* < 0.05). No differences between EFOV-US and TAPE-US method were found (*p* > 0.05). LMTU increased from 0° to 90° of hip flexion and 90° to 0° of knee flexion (*p* < 0.05).

The average ST LMTU for all combinations of hip and knee joint positions is presented in Table 3. There were statistically significant main effects for method (F_2,22_ = 3.66, *p* < 0.05, η^2^ = 0.31), hip (F_1,11_ = 955.87, *p* < 0.05, η^2^ = 0.98), and knee (F_2,22_ = 440.75, *p* < 0.05, η^2^ = 0.97). Post-hoc Tukey tests showed that LMTU measured using the EFOV-US and MODEL methods was greater than TAPE-US values (*p* < 0.05). No differences between the MODEL and EFOV-US method were found (*p* > 0.05). LMTU increased from 0° to 90° of the hip flexion and 90° to 0° of the knee flexion (*p* < 0.05).

The average SM LMTU for all combinations of hip and knee joint positions is presented in Table 4. There were statistically significant main effects for method (F_2,22_ = 41.17, *p* < 0.05, η^2^ = 0.79), hip (F_1,11_ = 601.47, *p* < 0.05. η^2^ = 0.98), and knee (F_2,22_ = 700.54, *p* < 0.05, η^2^ = 0.98). Post-hoc Tukey tests showed that the EFOV-US and MODEL technique yielded greater values than those measured using the TAPE-US technique (*p* < 0.05). Furthermore, the EFOV-US technique yielded lower LMTU compared with the MODEL technique (*p* > 0.05). LMTU increased from 0° to 90° of the hip flexion and 90° to 0° of the knee flexion (*p* < 0.05).

**Figure 4:**
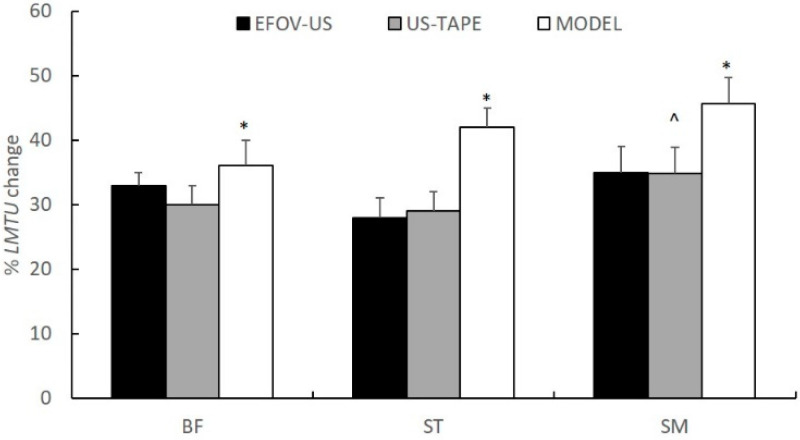
Mean (SD) maximum percentage change in muscle-tendon unit length of the biceps femoris long head (BFlh), semitendinosus (ST), and semimembranosus (SM) relative to the shortest muscle length (hip flexed 0° and knee flexed 90°), estimated using EFOV-US, TAPE-US, and MODEL methods (* significant different compared with EFOV-US and TAPE-US methods, collapsed across muscles ^ significantly different compared with BFlh and ST, collapsed across methods *p* < 0.05).

The maximum percentage change in LMTU for all muscles is presented in Figure 4. There were statistically significant main effects for the method (F_2,22_ = 5.87, *p* < 0.05, η^2^ = 0.74) and muscle (F_2,22_ = 13.05, *p* < 0.05, η^2^ = 0.35) on a maximum percentage LMTU change. The MODEL method showed greater overall changes in length compared with EFOV-US and TAPE-US methods (*p* < 0.05). No differences between EFOV-US and TAPE-US methods were observed (*p* > 0.05). Irrespective of the method, the SM LMTU showed approximately 5% greater change in length than BFlh and ST (*p* < 0.05). The BFlh and ST percentage length did not differ (*p* > 0.05).

## 4. Discussion

The main findings of this study were that: (a) LMTU and percentage change of LMTU of the hamstring muscles were greater using the MODEL compared with those measured using US-based methods, (b) LMTU differed between EFOV-US and TAPE-US techniques, and (c) the rate of length increase from shorter to longer length joint positions varied between methods and muscles. To our knowledge, this is the first study that reports LMTUs for the three hamstrings using three different methods.

The results of this study confirmed our first hypothesis as the MODEL method showed greater LMTUs of the BFlh (Table 2) and SM (Table 4) than the US-based methods. There are several factors that may have contributed to this finding. First, regression equations are based on anthropometric data obtained from cadavers and therefore, they cannot account for individual variations in morphology. Expression of LMTU as a percentage of thigh length assists in scaling LMTU to individuals. However, this may also be subject to error due to the direct measurement of femur length on skin surface. Second, the age of the cadavers is another limited factor of the MODEL technique as this may affect the absolute LMTU values [7]. The absence of differences between EFOV and MODEL techniques in ST LMTU may be attributed to a different definition of the distal origin of this muscle between the two methods. Specifically, the regression equations developed by Hawkins and Hull [8] assumed that the distal origin of the ST is the closest point where the muscle crosses the lateral tibia condyle while the remaining segment of the free tendon that wraps around the tibia and inserts into the fascia cruris was not taken into consideration. Based on our measurements, this segment represents approximately 40–45% of the ST distal free tendon and this might explain the absence of differences between EFOV-US and MODEL data.

In the present study, the MODEL technique yielded approximately 9–10% greater changes in length of all muscles compared with EFOV-US and TAPE-US methods (Figure 4). Differences in methodology used to obtain data may be responsible for this finding. First, regression equations were developed by modelling each muscle as a straight line and joints as simple hinges [8]. In contrast, both US-based methods take into consideration the curved path of the musculature, as the muscle-tendon unit does not have a constant thickness from distal to proximal ends. It is possible that changes in LMTU from shorter to longer lengths are associated with a reduction of the muscle path curvature (muscle thickness decreases) with less changes in linear distance between the two attachments. For the same reason, US scanning of the distal tendons as they wrap around the bones may yield less changes in tendon/aponeurosis length than simply assuming that the tendons move linearly (as a straight line) as the tibia moves. Second, it is possible that some changes in distal and proximal origins of the muscles were not visible in the US field of view, thus underestimating the overall changes in LMTU as a function of hip and knee angle changes. Third, MODEL equations yield standardised length changes, without taking into consideration variations in muscle-tendon architecture between individuals.

The second finding of this study was that the two US-based methods yielded different LMTU results, depending on the muscle under examination. Hence, the second hypothesis is partly confirmed. In particular, the BFlh LMTU (Table 2) did not differ between the two techniques while the ST (Table 3) and SM (Table 4) LMTUs measured using the TAPE-US method were consistently lower than EFOV measures. There are several factors that may have contributed to these results. First, the BFlh distal origin (fibula) can be more easily palpated and marked on the skin compared with the identification of the distal origin of the SM and ST (lateral tibial condyle area). Second, the BFlh muscle path is generally easier to identify on the skin compared with the ST and SM paths, which are located very close to one another. Third, the EFOV method is not affected by the adipose fat tissue and fascia underneath the skin. The thickness of this tissue varies along muscle length and it also shows considerable inter-individual variability. This might have contributed to the lower values of the TAPE-US method as opposed to the EFOV method. Finally, identification of the proximal attachment may also vary between the two techniques, specifically, proximally all hamstrings run deep underneath the gluteus maximus and magnus adductor. Placing a mark on the skin over the ischial tuberosity provides an estimate of the actual location of the ischial tuberosity but it cannot take into account the vertical insertion of the muscles to the ischial tuberosity.

Quantification of hamstring LMTUs using US-assisted methods displays a number of limitations. Firstly, both techniques used in this study are based on longitudinal two-dimensional US images of each muscle. This does not take into account the three-dimensional shape of each muscle and the values are highly specific to the path of the scanning probe. In the present study, the scanning path was chosen such that the fascicle orientation and superficial and deep aponeuroses of the muscle were visible. Secondly, the identification of the two ends of the muscle was subject to some assumptions. Distally, it was assumed that the BFlh and SM arise from the fibula and tibia, respectively. While the image clearly indicates the tendon path, precise identification of tendon insertion is not always possible. Of the three muscles, identification of the SM path was the most problematic and distally, the SM fibers and tendon are easily identified. However, identification of the most proximal part is difficult as the muscle lies slightly medially and deeper to the ST and to the adductor magnus (most proximally). A common limitation of the US techniques is that actual length measurements can be distorted by the orientation of the probe and it is subject to perspective and parallax measurement errors [25,26]. A limitation of this study is that all measurements were performed by the same operator. While reliability of measurements was moderate to high, between operators’ reliability was not examined.

The third hypothesis of this study was that there will be differences in length between the individual hamstring muscles at various joint configurations. All methods applied in this study indicated that the SM LMTU displayed the highest relative change compared with BFlh and ST (Figure 4). This is not in line with previous studies in cadavers [12] or geometric modelling [11], which reported a greater change of ST length compared with BFlh and SM. Differences in measurement method (cadaveric vs modelling vs US) and assessment protocol (joint range of motion) may account for these variations. There are various factors that could have contributed to the greater change in LMTU by SM than the remainder hamstrings. Since SM and BFlh are pennated muscles, they may experience greater change in length during passive motion than the fusiform ST [27]. Differences in tendon properties and moment-arms may also influence length changes, but the exact contribution is still unclear [5].

Reduced resistance to stretch and an altered muscle force—length capacity are important factors for hamstring muscle function and injury [2,3,14,28]. Quantification of these factors partly depends on an accurate measurement of hamstring LMTU. The results of this study indicate that modelling the hamstrings by applying regression equations (at least the ones used in this study) in combination with joint angle data not only provide standardised (similar for everyone) but also greater changes in length compared with US-based techniques. This may lead to erroneous conclusions regarding hamstring mechanical properties, especially when LMTU estimates are combined with US measurements of fascicle or tendon lengths. The use of US techniques overcome these limitations as they allow for a direct assessment of individual LMTU. However, a limitation of current US techniques is that they allow LMTU assessment only under static joint positions. The TAPE-US technique is easier to apply compared with EFOV-US while it can be used in most diagnostic US systems. The present findings suggest that the US-TAPE method provides lower LMTUs than EFOV-US, thus indicating that this technique should be used with caution. Finally, EFOV-US takes into consideration the curvature of the specific muscle under examination, and, hence, it overcomes some of the limitations of the US-TAPE method.

Based on the above, there are some practical implications of the present findings. First, when the aim is to measure length changes under static conditions, such as passive stretch or examination of muscle morphology at different joint angles, the use of US appears more promising. This is because US techniques provide an easy, direct, and non-invasive measurement of hamstring length. Amongst the two US methods, the EFOV-US seems more promising as it takes the curved path of each muscle into consideration. Finally, studies utilising joint angle data and modelling equations to predict hamstring muscle length should be aware that it provides greater absolute and relative muscle length values than US-based methods. Future studies could examine whether quantification of muscle length changes during dynamic movement conditions based on kinematic data is best achieved when using static length values directly obtained using US instead of length values predicted using regression model equations.

A limitation of this study is that the results are based on a relatively small sample size. Since differences between variables were statistically significant and with an adequate effect size, this effect is likely to be minimal.

## 5. Conclusions

The specific regression equations applied in this study tended to overestimate hamstring length compared with US-based measurements. Using the EFOV-US method yielded greater ST and SM length than the TAPE-US method. Irrespective of method, the ST showed the greater length and the SM the greater change in length as a result of changing hip and knee joint angles. Studies examining hamstring mechanics may reach different conclusions depending on the method used to measure muscle-tendon lengths. 

## Figures and Tables

**Table 1:** Reliability values for length of each hamstring using extended field-of-view ultrasonography (EFOV-US) and the ultrasound tape (TAPE-US) method (*n* = 7).

	EFOV-US			TAPE-US		
	ICC_2,1_	SEM	SEM (%)	ICC_2,1_	SEM	SEM (%)
Semitendinosus						
Hip–Knee Angle (°)						
0–0	0.99	0.61	1.44	0.90	0.88	2.17
0–45	0.90	0.65	1.61	0.91	0.73	1.94
0–90	0.82	0.61	1.57	0.85	0.65	1.87
90–0	0.87	1.01	2.10	0.88	1.45	3.16
90–45	0.88	0.99	2.16	0.88	1.40	3.38
90–90	0.91	0.81	1.83	0.94	1.81	4.63
Biceps femoris						
Hip–Knee Angle (°)						
0–0	0.98	0.58	1.75	0.93	0.60	2.63
0–45	0.95	0.90	2.95	0.89	0.92	2.64
0–90	0.96	0.73	2.52	0.93	1.01	2.92
90–0	0.93	0.92	2.36	0.91	1.11	2.14
90–45	0.93	0.93	2.53	0.88	0.92	2.25
90–90	0.90	0.89	2.53	0.89	1.01	2.43
Semimembranosus						
Hip–Knee Angle (°)						
0–0	0.92	0.78	2.13	0.95	0.63	1.81
0–45	0.84	0.62	1.77	0.94	0.94	2.94
0–90	0.88	0.50	1.50	0.85	1.03	3.51
90–0	0.90	0.87	2.06	0.91	1.01	2.52
90–45	0.91	0.87	2.17	0.87	0.93	2.61
90–90	0.92	0.77	2.01	0.86	1.13	3.39

ICC: Intraclass correlation coefficient, SEM = standard error of measurement.

**Table 2:** Mean (±SD) group values of the BFlh length (cm) at six combinations of hip and knee flexion angles, assessed using three different methods (* statistically significant different compared with TAPE-US and EFOV-US method, ^ values collapsed across methods and knee joint positions significantly different compared with a 0° hip flexion angle, # values collapsed across methods, and hip joint angles significantly different compared with a 0° knee flexion angle, *p* < 0.05).

	Method		
Hip–Knee Angle (°)	TAPE-US (cm)	EFOV (cm)	MODEL (cm)
0–0	36.04 ± 3.07	34.96 ± 4.34	42.22 ± 4.21 *
0–45	34.14 ± 3.17	32.58 ± 4.19	39.32 ± 3.92 *
0–90 #	32.58 ± 3.52	30.92 ± 3.91	36.42 ± 3.63 *
90–0 ^	41.62 ± 3.26	40.95 ± 4.10	49.80 ± 4.97 *
90–45 ^	39.20 ± 3.39	38.52 ± 4.31	46.90 ± 4.68 *
90–90 ^#	36.83 ± 3.40	37.08 ± 4.07	44.00 ± 4.39 *

**Table 3:** Mean (± SD) group values of ST length (cm) at six combinations of hip and knee flexion angles, assessed using three different methods (* statistically significant different compared with the TAPE-US and EFOV-US method, ^ values collapsed across methods and knee joint positions significantly different compared with a 0° hip flexion angle, # values collapsed across methods, and hip joint angles significantly different compared with a 0° knee flexion angle, *p* < 0.05).

	Method		
Hip–Knee Angle (°)	TAPE-US (cm)	EFOV (cm)	MODEL (cm)
0–0	41.68 ± 2.97	40.69 ± 4.37	39.76 ± 3.97
0–45	36.35 ± 2.68	38.16 ± 4.75	36.54 ± 3.65
0–90 #	33.89 ± 2.45	36.28 ± 5.28	33.31 ± 3.32
90–0 ^	43.68 ± 2.45	46.12 ± 4.78 *	47.27 ± 4.72 *
90–45 ^	39.90 ± 2.87	43.47 ± 5.19 *	44.04 ± 4.40 *
90–90 ^#	37.56 ± 3.07	41.68 ± 5.19 *	40.81 ± 4.07 *

**Table 4:** Mean (± SD) group values of SM length (cm) at six combinations of hip and knee flexion angles, assessed using three different methods (* statistically significant different compared with the TAPE-US and EFOV-US method, ^ values collapsed across methods and knee joint positions significantly different compared with a 0° hip flexion angle, # values collapsed across methods, and hip joint angles significantly different compared with a 0° knee flexion angle, *p* < 0.05).

	Method		
Hip–Knee Angle (°)	TAPE-US (cm)	EFOV (cm)	MODEL (cm)
0–0	33.25 ± 2.63	35.13 ± 3.46 *^	41.37 ± 4.13 *
0–45	30.67 ± 2.39	33.06 ± 3.99 *^	37.35 ± 3.72 *
0–90 #	28.22 ± 2.18	31.33 ± 4.07 *^	33.32 ± 3.33 *
90–0 ^	38.00 ± 3.86	41.68 ± 1.74 *^	47.27 ± 4.72 *
90–45 ^	34.20 ± 2.72	39.28 ± 1.81 *^	44.04 ± 4.40 *
90–90 ^#	31.87 ± 2.97	36.83 ± 3.29 *^	40.81 ± 4.07 *

## Data Availability

Data supporting reported results can be found at: http://dx.doi.org/10.17632/rrpbmtbg6t.1 (accessed on 1 March 2021).
